# Case of an Abscess in the Lower Part of the Belly Which Communicated with the Intestine, and Terminated Successfully

**Published:** 1790

**Authors:** George Grant

**Affiliations:** Surgeon


### IV. *Case of an Abscess in the lower Part of the Belly, which communicated with the Intestine, and terminated successfully. By Mr.* George Grant, *Surgeon. Communicated to Dr.* Simmons *by* Everard Home, *Esq. F. R. S.*

THE following case, although it cannot be considered as new, is certainly uncommon, and is an additional fact in proof of the opinion inculcated by Mr. Hunter, in his lectures, that wounds or abscesses may open a communication with an intestine, after previous inflammation has united the surrounding parts and excluded the general cavity of the belly, and may be successfully treated.

Mrs. Apleton, a soldier's wife of the fifty-ninth regiment, complained of violent pain in the lower part of the belly. Upon examination, there was found to be a hard tumour situated in the left iliac region where the colon passes down to get into the pelvis. It was of the size of a pigeon's egg, very tense, and had a considerable degree of inflammation surrounding its base. As it was too far advanced to give any hopes of its being resolved either by internal medicines or by topical applications, it was allowed allowed to come forward, and when matter was formed, and an evident fluctuation felt, it was opened with a lancet. The contents that were evacuated were not true pus, but an ichorous discharge tinged with blood.

The lips of the wound did not put on an healthy appearance, but became a foul ulcer, and the matter did not change its appearance.

On the eighth day after it was opened she was seized with a diarrhœa, which gradually gave way to the use of proper medicines, but left her much weakened.

On the tenth day, upon removing the dressings, a fœtid matter, mixed with fæces, was discharged; and there was a sensible smell from the escape of putrid air. Upon introducing a probe, it passed a considerable way, giving pain and uneasiness along the course of the intestine, and producing sickness; so that it was evident the abscess had opened into the intestine, and from this period the fæces were in part evacuated along with the matter.

The patient was supported by strengthening medicines: her bowels were kept gently open, and the external wound was kept clean by changing the poultices frequently. Under this treatment the ulcer put on a better appearance, the matter became lefs offenfive, and lefs tinged by the fæces, till at laft no fæces or offenfive fmell could be perceived at all, and at prefent there is only a fmall fuperficial fore, which will probably foon heal, like any other fimilar fore on the fkin.

*Gibraltar,*

July 21, 1789.

